# Increased inflammatory markers correlate with liver damage and predict severe COVID-19: a systematic review and meta-analysis 

**Published:** 2020

**Authors:** Nasrin Amiri-Dashatan, Mehdi Koushki, Fatemeh Ghorbani, Nosratollah Naderi

**Affiliations:** 1 *Proteomics Research Center, Faculty of Paramedical Sciences, Shahid Beheshti University of Medical Sciences, Tehran, Iran*; 2 *Department of Clinical Biochemistry, School of Medicine, Zanjan University of Medical Sciences, Zanjan, Iran*; 3 *Department of Clinical Biochemistry, Faculty of Medicine, Tehran University of Medical Sciences, Tehran, Iran*; 4 *Gastroenterology and Liver Diseases Research Center, Research Institute for Gastroenterology and Liver Diseases, Shahid Beheshti University of Medical Sciences, Tehran, Iran *

**Keywords:** COVID-19, Coronavirus, Inflammatory markers, CRP, TNFα, IL-6, Meta-analysis

## Abstract

**Aim:**

This study aimed to determine whether patients with elevated CRP, TNFα, and IL-6 levels may be at increased risk for severe infection and liver damage of COVID-19.

**Background:**

The COVID-19 outbreak is a serious health problem to human beings. The evidence suggests that inflammatory markers related to liver damage increase in severe forms of COVID-19 compared to mild cases.

**Methods:**

The electronic databases ISI Web of Science, EMBASE, and Cochrane Library were comprehensively searched for articles published up to May, 2020. Data from each identified study was combined using the random effects model to estimate standardized mean difference (SMD) and 95% confidence intervals (95% CIs). Sensitivity and publication bias were also calculated.

**Results:**

Totally, 23 studies were included in this meta-analysis comprising 4313 patients with COVID-19. The random effects results demonstrated that patients with severe COVID-19 had significantly higher levels of CRP [SMD = 3.26 mg/L; (95% CI 2.5, 3.9); p<0.05; I2 = 98.02%; PHeterogeneity = 0.00], TNFα [SMD = 1.78 ng/mL; (95% CI 0.39, 3.1); p=0.012; I2 = 98.2%; PHeterogeneity = 0.00], and IL-6 [ SMD = 3.67 ng/mL; (95% CI 2.4, 4.8); p<0.05; I2 = 97.8%; PHeterogeneity = 0.00] compared with those with the mild form of the disease. Significant heterogeneity was present. No significant publication bias was observed in the meta-analysis. Sensitivity analyses showed a similar effect size while reducing the heterogeneity.

**Conclusion:**

The data suggests that enhanced inflammation may be associated with COVID-19-related liver damage, possibly involving inflammatory marker-related mechanisms.

## Introduction

 COVID-19 was first identified in December, 2019, in Wuhan, China and was named Severe Acute Respiratory Syndrome 2 (SARS-CoV-2) by the International Committee on the Taxonomy of Viruses (ICTV) (1, 2). This pneumonia was then registered as COVID-19 (coronavirus disease 2019) by the World Health Organization (3). The outbreak of COVID-19 was a major threat to global public health (4). As of May 21, 2020, a total of 4,970,000 cases were confirmed in 213 countries and territories including nearly 322,144 deaths (5). Patients with COVID-19 display a ranged of mild symptoms, including dry cough, fever, and fatigue; in most instances, these symptoms improve, yet some progress to Acute Respiratory Distress Syndrome (ARDS), organ failure, septic shock, and ultimately death (6). A growing body of evidence indicates that systemic inflammation promotes the progression of COVID-19 following the release of pro-inflammatory cytokines (7, 8). Therefore, it appears that strategies based on the inhibition of inflammatory responses can be of interest in the treatment of severe COVID-19. Several studies have reported the association between inflammatory markers of C-reactive protein (CRP), tumor necrosis factor (TNF-), and interleukin-6 (IL-6) and the high risk of developing severe COVID-19 (9, 10). CRP, which is the most commonly used marker of systemic inflammation, is increased in many respiratory viral infections (11).

**Figure 1:**
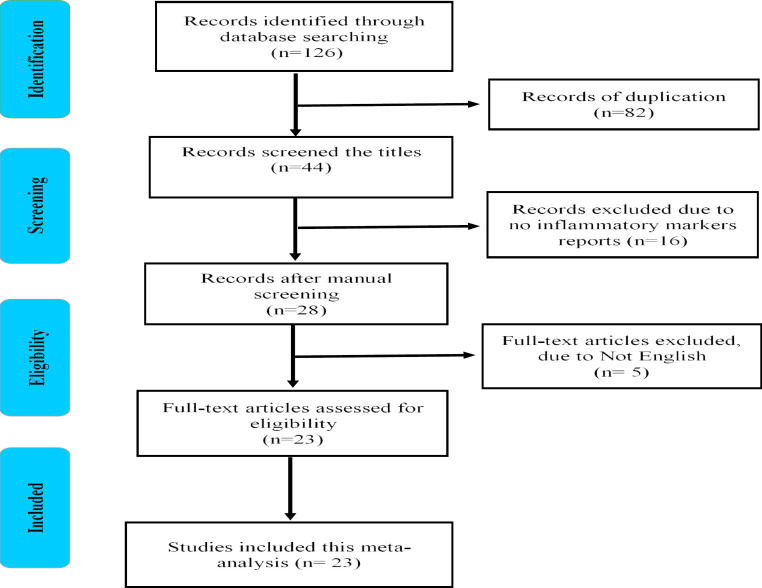
Flow chart showing the number of articles detected at each stage of the search

 TNF- and IL-6 are two key cytokines released in large amounts during inflammation that most studies have reported as being potential predictors of severe COVID-19 (12, 13). Elevated levels of CRP, TNF-, and IL-6 are involved in COVID-19 pathogenesis and may act as potential biomarkers for the management of the severity of the disease (14). Intriguingly, studies on the inflammatory markers in patients with COVID-19 have reported conflicting results. Therefore, the objective of the present study was to investigate the association of inflammatory markers with severity in patients presenting with COVID-19 through a systematic review and meta-analysis. 

## Methods

**Data sources and search strategy**

This systematic review and meta-analysis were performed according to the Preferred Reporting Items for Systematic Reviews and Meta-analyses (PRISMA) guidelines. Studies published up to May, 2020 were collected through a search of the ISI web of sciences, PubMed, EMBASE, Scopus, Google Scholar, and Cochrane Library databases for observational studies that reported the association between inflammatory marker levels and COVID-19 severity. The following keywords were used in the search: (“Inflammatory Markers” OR “Inflammatory Cytokines” AND “COVID-19” OR “SARS-Cov19” OR “Severe Acute Respiratory Syndrome Coronavirus” OR “Coronavirus”), (“CRP” OR “C-reactive Protein” AND “COVID-19” OR “SARS-CoV19” OR “Severe Acute Respiratory Syndrome Coronavirus” OR “Coronavirus”), (“TNF-” OR “Tumor Necrosis Factor-” AND “COVID-19” OR “SARS-CoV19” OR “Severe Acute Respiratory Syndrome Coronavirus” OR “Coronavirus”), and (“IL-6” OR “Interleukin-6” AND “COVID-19” OR “SARS-Cov19” OR “Severe Acute Respiratory Syndrome Coronavirus” OR “Coronavirus”). The reference lists of searched articles were also reviewed to include additional relevant studies.

**Study selection criteria**

One author (NAD) conducted the initial screening of the paper titles and abstracts identified in the first screening step. Two authors, (NAD) and (MK), independently reviewed the full text of the selected papers for final inclusion. The inclusion criteria included: (1) being an original article; (2) observational studies with a retrospective design; (3) estimated inflammatory markers (including CRP, TNF- and IL-6) related to COVID-19, and (4) published in English.

**Data extraction and quality assessment**

Data was extracted by NAD and independently checked by MK for precision. From every study, data on the first author’s surname, publication year, study design, country of study, gender of subjects, sample size, mean (SD) age, and the serum levels of CRP, TNF- and IL-6 in mild and severe cases of COVID-19 was extracted. Interquartile ranges (IQR) were converted to means. Two investigators independently evaluated the quality of the included studies using the Newcastle–Ottawa Scale (NOS). The selection, comparability, and exposure of each study were broadly assessed and studies were assigned a score from zero to nine. A score of four to six was regarded as moderate quality, and studies with scores ≥7 were considered to be of high quality.

**Statistical analysis**

All statistical analyses were performed by CMA (comprehensive meta-analysis) V2 software (Biostat, NJ, USA) (15). A *p*-value < 0.05 was considered statistically significant. Forest plots of binary data of CRP, TNF-, and IL-6 in patients of mild and severe COVID-19 infection were built using standardized mean difference (SMD) and 95% confidence intervals (95%CI). For each group of variables, a random-effects model was used to calculate the SMD of the severity of COVID-19 infection. The heterogeneity among studies was estimated by the Q test (*p*<0.1) and the I^2^ statistics, characterized by the percentage of the total variation in effect size that can be associated with heterogeneity. Values > 50% and 70% were considered as moderate and high heterogeneity, respectively. Sensitivity was estimated by sequentially excluding one study in each turn to evaluate the robustness of the results. Publication bias was evaluated by the Begg’s rank correlation test and Egger’s regression asymmetry test by the funnel plot (16, 17). Where publication bias was significant, the number of missing studies was estimated with the trim-and-fill function and visualized with a funnel plot. 

## Results

**Study selection and study characteristics**

The electronic database searches identified a total of 126 records with 82 duplicate studies which were excluded from this meta-analysis. After the initial screening of title and abstract, 44 articles were retrieved for further screening. An additional 16 articles were removed due to incomplete reports. Of the remaining 28 articles, 5 were removed for not being in English. After excluding these articles, the 22 articles that met the inclusion criteria were included in this meta-analysis (Fig. 1). 

The characteristics extracted from the studies included in this meta-analysis are summarized in Table 1. All included studies came from China and were published in 2020. Twenty-three observational studies involving 2454 mild cases and 1859 severe cases of COVID-19 were identified. All studies were conducted with both men and women. The diagnosis of COVID-19 was based on real-time RT-PCR in all studies. CRP values were reported in most of the included studies, but only 5 (22.72%) studies evaluated the TNF- marker, and IL-6 was reported in 9 studies. In addition, 3 studies reported CRP, TNF-, and IL-6. Using the Newcastle–Ottawa Scale, the methodological quality of the studies was scored at ≥7 (Table 2).

**Table 1:** Extracted characteristics of included studies in the systematic review and meta-analysis

*Athour's name/ Country (ref)	Samples size	Gender (M/ F)	Age (year)(Mean ± SD)	Inflammatory cytokines		
				CRP (Mean ± SD)	TNF- (Mean ± SD)	IL-6 (Mean ± SD)
Cai J et al. (2020)/ China (13)	Overall:298Mild: 240Severe: 58	Overall: 145/153Mild: 106/134Severe: 39/19	Overall: 47.2 ± 4.6Mild: 42.25 ± 4.16Severe: 61.75 ± 2.5	Overall: 134.4 ± 35.9 Mild: 102.6 ± 30.3 Severe: 348.2 ± 99.4	-	Overall: 11.46 ± 2.54Mild: 9.42 ± 2.1Severe: 28.39 ± 8.52
Cao W et al. (2020)/ China (18)	Overall:128Mild: 107Severe: 21	Overall: 65/63Mild: 48/59Severe: 12/9	-	Overall: 35.83± 17.88 Mild: 29.85 ± 32.9 Severe: 37.92 ± 29.57	-	-
Jin X et al. (2020)/ China (19)	Mild: 577 Severe: 74	Mild: 294/283 Severe: 37/37	Mild: 45.09 ± 14.45 Severe: 46.14 ± 14.19	Mild: 9.48 ± 2.82 Severe: 15.03 ± 3.19	-	-
Mo P et al. (2020)/ China (20)	Overall: 155Mild: 70Severe: 85	Overall: 86/69Mild: 31/39Severe: 55/30	Overall: 54 ± 4Mild: 45.75 ± 3.5Severe: 60.75 ± 3.16	Overall: 39 ± 9.66Mild: 25.75 ± 6.16Severe: 55 ± 14	-	Overall: 50. 75 ± 13.16Mild: 28 ± 8Severe: 81 ± 22.33
Pan L et al. (2020)/ China (21)	Overall: 204 Mild: 105 Severe: 99	Overall: 107/97 Mild: 53/52 Severe: 54/45	Overall: 54.9 ± 15.4Mild: 55.2 ± 14.7Severe: 54.6 ± 16.1	Overall: 57.5 ± 54.1Mild: 50.1 ± 57.3Severe: 63.7 ± 50.8	-	-
Qian GQ et al. (2020)/ China (22)	Overall: 9Mild: 82Severe: 9	Overall: 37/54Mild: -Severe: -	Overall: -Mild: 47.32 ± 3.45Severe: 66.5 ± 6.5	Overall: -Mild: 6.16 ± 1.65Severe: 44.29 ± 22.72	-	-
Qin C et al. (2020)/ China (9)	Overall: 452 Mild: 166 Severe: 286	Overall: 235/217 Mild: 80/86 Severe: 155/131	Overall: 57.5 ± 3.33 Mild: 52.3 ± 3.45 Severe: 60.5 ± 3.0	Overall: 49.3 ± 13 Mild: 33.57 ± 8.58 Severe: 59.97 ± 13.71	Overall: 8.75 ± 0.66Mild: 8.52 ± 0.58Severe: 9.02 ± 0.75	Overall: 23.82 ± 6.85Mild: 17.9 ± 6.2Severe: 28.6± 7.5
Ruan Q et al. (2020)/ China (23)	Overall: 150 Mild: 82 Severe: 68	Overall: - Mild: 53/29 Severe: 49/19	Overall: - Mild: 56.25 ± 6.16 Severe: 57.5 ± 16.5	Overall: - Mild: 34.1 ± 54.5 Severe: 126.6 ± 106.3	Overall: - Mild: 6.8 ± 3.61 Severe: 11.4± 8.51	-
Chen G et al. (2020)/ China (12)	Overall::21Mild: 10Severe: 11	Overall::17/4Mild: 7/3 Severe: 10/1	Overall: 56.3 ± 14.3Mild: 51.4 ± 13.7Severe: 63.9 ± 9.6	Overall: 97.5 ± 66.4Mild: 51.4 ± 50.8Severe: 135.2 ± 52.4	Overall: 9.4 ± 3.0Mild: 7.5 ± 1.6Severe: 10.9 ± 3.0	Overall: 51.2 ± 59.4Mild: 18.8 ± 13.9Severe: 73.8 ± 67.9
Chen T et al. (2020)/ China (24)	Overall: 274Mild: 161Severe: 113	Overall: 171/102Mild: 88/73Severe: 83/30	Overall: 59.5 ± 4.33Mild: 51.25 ± 4.83Severe: 68.75 ± 2.5	Overall: 59.6 ± 15.73Mild: 64.5 ± 7.85Severe: 115.87 ± 16.55	Overall: 9.1± 0.86Mild: 8.02 ± 0.48Severe: 12.45 ± 1.5	Overall:25.47± 7.61Mild: 14.05 ± 3.7Severe: 81.6 ± 18.53
Gao Y et al. (2020)/ China (10)	Overall: 43Mild: 28Severe: 15	Overall: - Mild: 17/11Severe: 9/6	Overall: -Mild: 42.96 ± 14.00Severe: 645.2 ± 7.68	Overall: -Mild: 18.76 ± 22.20Severe: 39.37 ± 27.68	-	Overall: - Mild: 12.62 ± 4.76Severe: 38.6 ± 9.05
Liu W et al. (2020)/ China (25)	Overall: 78Mild: 67Severe: 11	Overall: 39/39Mild: 32/35Severe: 7/4	Overall: 41.5 ± 4Mild: 36.75 ± 2.25Severe: 63.25 ± 4.75	Overall: 15.12 ± 5.61Mild: 14.05 ± 7.8Severe: 39.22 ± 12.62	-	-
Fan Z et al. (2020)/ China	Overall: 148Mild: 73Severe: 75	Overall: 73/75Mild: 28/45Severe: 47/28	-	Overall: -Mild: 6.86 ± 1.5Severe: 26.14 ± 5.12	-	-
Zhou F et al. (2020)/ China (26)	Overall: 191Mild: 137Severe: 54	Overall: 119/72Mild: 81/56 Severe: 38/16	Overall: 56.25 ± 3.5Mild: 51.75 ± 2.16Severe: 69.25 ± 3.25	-	-	Overall: 7.72 ± 0.91Mild: 6.15 ± 0.48Severe: 10.97 ± 1.72
Zhang JJ et al. (2020)/ China (27)	Overall: 140Mild: 82Severe: 58	Overall: 71/69Mild: 38/44Severe: 33/25	Overall: 56.6 ± 10.33Mild: 51.75 ± 8.66Severe: 60 ± 15.5	Overall: 37.07 ± 9.15Mild: 29.75 ± 7.1Severe: 50.72 ± 16.62	-	-
Zhang X et al. (2020)/ China (28)	Overall: 645Mild: 72Severe: 573	Mild: 33/39Severe: 295/278	Mild: 34.9 (14.2)Severe: 46.65 ± 13.82	Mild: 3.75 ± 1.43Severe: 10.67 ± 3.15	-	-
Deng Y et al. (2020)/ China (29)	Mild: 116Severe: 109	Mild: 51/73Severe: 65/36	Mild: 42.5 ± 4Severe: 68.5 ± 2	Mild: 7.32 ± 3.46Severe: 105.94 ± 22.54	-	-
Wan S et al. (2020)/ China (30)	Overall: 135Mild: 95Severe: 40	Overall: 72/63 Mild: 52/43Severe: 21/19	Overall: 46.25 ± 3.16Mild: 42.5 ± 2.66Severe: 59.25 ± 3.5	Overall: 18.72 ± 8.08Mild: 12.1 ± 4.86Severe: 92.75 ± 20.9	-	-
Xie H et al. (2020)/ China (31)	Overall: 79Mild: 51Severe: 28	Overall: 44/34Mild: 26/25Severe: 18/10	Overall: 58.5 ± 3Mild: 57.5 ± 5Severe: 60.82 ± 4.32	(N= 16) Overall: 32.75 ± 24.15Mild: 10.55 ± 4.55Severe: 69.05 ± 12.5	-	-
Yang B et al. (2020)/ China (32)	Overall: 18 Mild: 12 Severe: 6	Overall: 9/9 Mild: 7/5 Severe: 2/4	Overall: 49.5 ± 10.5 Mild: 40.25 ± 6.25 Severe: 58 ± 6.5	Overall: 20.7 ± 8.13Mild: 14.07 ± 7.42Severe: 34.65 ± 13.8	-	-
Wu C et al. (2020)/ China (33)	Overall: 84 Mild: 40 Severe: 44	Overall: 60/24 Mild: 31/9 Severe: 29/15	Overall: 59 ± 3.16 Mild: 49.27 ± 4.12 Severe: 67.82 ± 3.92	Overall: 89.46 ± 18.82 Mild: 71.45 ± 23.55 Severe: 96.56 ± 28.86	-	Overall: 7.82 ± 0.87 Mild: 6.05 ± 0.46 Severe: 10.57 ± 1.86
Zhou B et al. (2020)/ China (34)	Mild: 26 Severe: 8	Mild: 12/14 Severe: 5/3	Mild: 33.25 ± 2.75 Severe: 51.25 ± 2.25	Mild: 23.42 ± 7.85 Severe: 74.88 ± 20.09	-	-
Wang Z et al. (2020)/ China (35)	Overall: 69 Mild: 55 Severe: 14	Overall: 32/37 Mild: 25/30 Severe: 7/7	Overall: 45.25 ± 4.5 Mild: 39.25 ± 4.75 Severe: 70 ± 3.75	Overall: 20.54 ± 10.55Mild: 13.85 ± 4.49Severe: 79.46 ± 14.26	Overall (N= 43) Mild (N= 36) Severe (N= 7)	
					Overall: 2.1 ± 0.1Mild: 1.95 ± 0.1Severe: 2.13 ± 0.11	Overall: 10.58 ± 3.97Mild: 7.56 ± 1.99Severe: 74.83 ± 31.83

*Study design for all studies are Retrospective

**Table 2:** Methodological quality of identified studies according to Newcastle-Ottawa Scale (NOS) check list

Eligible studies	Is thedefinitionadequate?	Representativenessof the cases	Selectionof controls	Definitionof controls	Comparabilityof both groups	Ascertainmentof diagnosis	Sameascertainmentmethod for bothgroups	Nonresponserate	Totalscores
Cai J et al.	*	*	*	*	*	*	*	*	8
Cao W et al.	*	*	*	*	*	*	*	*	8
Mo P et al.	*	*	*	*	*	*	*	*	8
Qian et al.	*	*	*	*	-	*	*	*	7
Qin C et al.	*	*	*	*	**	*	*	*	9
Ruan Q et al.	*	*	*	*	-	*	*	*	7
Chen G et al.	*	*	*	*	**	*	*	*	9
Chen T et al.	*	*	*	*	**	*	*	*	9
Gao Y et al.	*	*	*	*	-	*	*	*	7
Liu W et al.	*	*	*	*	*	*	*	*	8
Zhang et al.	*	*	*	*	**	*	*	*	9
Zhang et al.	*	*	*	*	-	*	*	*	7
Deng Y et al.	*	*	*	*	-	*	*	*	7
Wan S et al.	*	*	*	*	*	*	*	*	8
Xie H et al.	*	*	*	*	*	*	*	*	8
Yang B et al.	*	*	*	*	**	*	*	*	9
Wu C et al.	*	*	*	*	-	*	*	*	7
Zhou B et al.	*	*	*	*	-	*	*	*	7
Wang Z et al.	*	*	*	*	*	*	*	*	8
Jin X et al.	*	*	*	*	-	*	*	*	7
Pan L et al.		*	*	*	-	*	*	*	7
Fan Z et al.	*	*	*	*	-	*	*	*	7
Zhou F et al.	*	*	*	*	-	*	*	*	7

**Figure 2:**
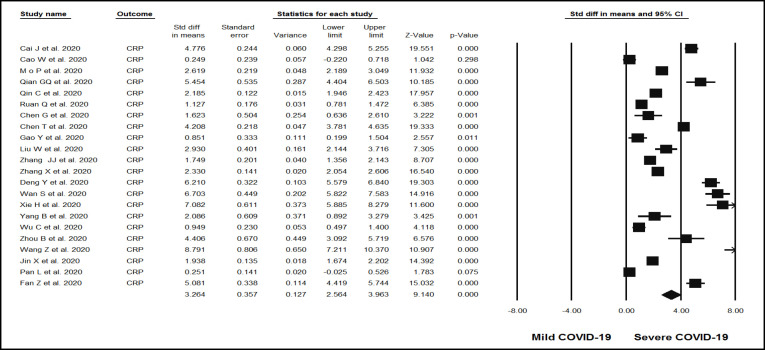
Forest plots assessing standardized mean difference (SMD) and 95% confidence intervals for the association between circulating levels of CRP with severity of n-COVID-19 in hospitalized patients. Meta-analysis was performed using a random-effects model with inverse variance weighting

**Meta-analysis of inflammatory markers and the severity of COVID-19**

**Figure 3:**
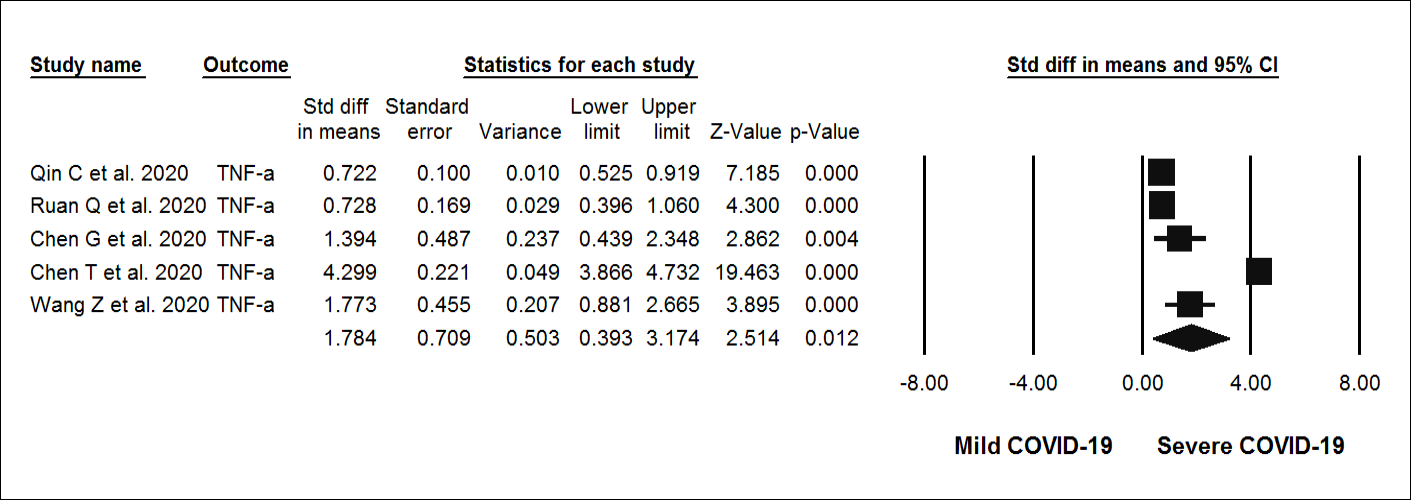
Forest plots assessing standardized mean difference (SMD) and 95% confidence intervals for the association between circulating levels of TNF-  with severity of n-COVID-19 in hospitalized patients. Meta-analysis was performed using a random-effects model with inverse variance weighting

**Figure 4:**
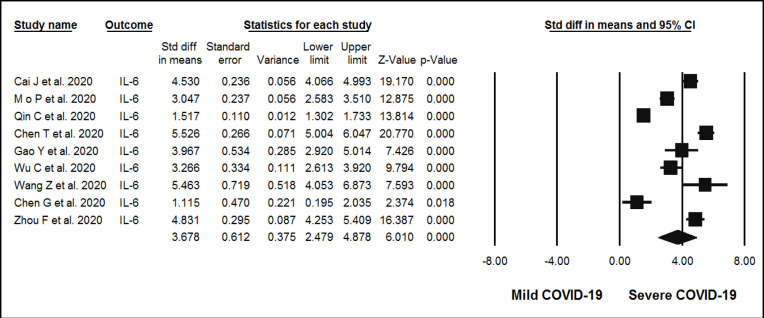
Forest plots assessing standardized mean difference (SMD) and 95% confidence intervals for the association between circulating levels of IL-6 with severity of n-COVID-19 in hospitalized patients. Meta-analysis was performed using a random-effects model with inverse variance weighting

Across the 23 evaluated observational studies, the association between the levels of inflammatory markers and the severity of COVID-19 and the levels of CRP, TNF-, and IL-6 was significantly high in the group with severe COVID-19 compared to the mild group. The random-effect summary of SMD for CRP [SMD = 3.26 mg/L; (95% CI 2.5, 3.9); *p*<0.05; I^2^ = 98.02%; *P*_Heterogeneity_ = 0.00] (Fig. 2), TNF- [SMD = 1.78 ng/mL; (95% CI 0.39, 3.1); *p*=0.012; I^2^ = 98.2%; P_Heterogeneity_ = 0.00] (Fig. 3), and IL-6 [ SMD = 3.67 ng/mL; (95% CI 2.4, 4.8); *p*<0.05; I2 = 97.8%; P_Heterogeneity_ = 0.00] (Fig. 4) were directly associated with risk of severe COVID-19. Statistical significant heterogeneity across the selected studies was evidenced by the I^2^ statistics for inflammatory markers and severity of COVID-19. 

**Figure 5:**
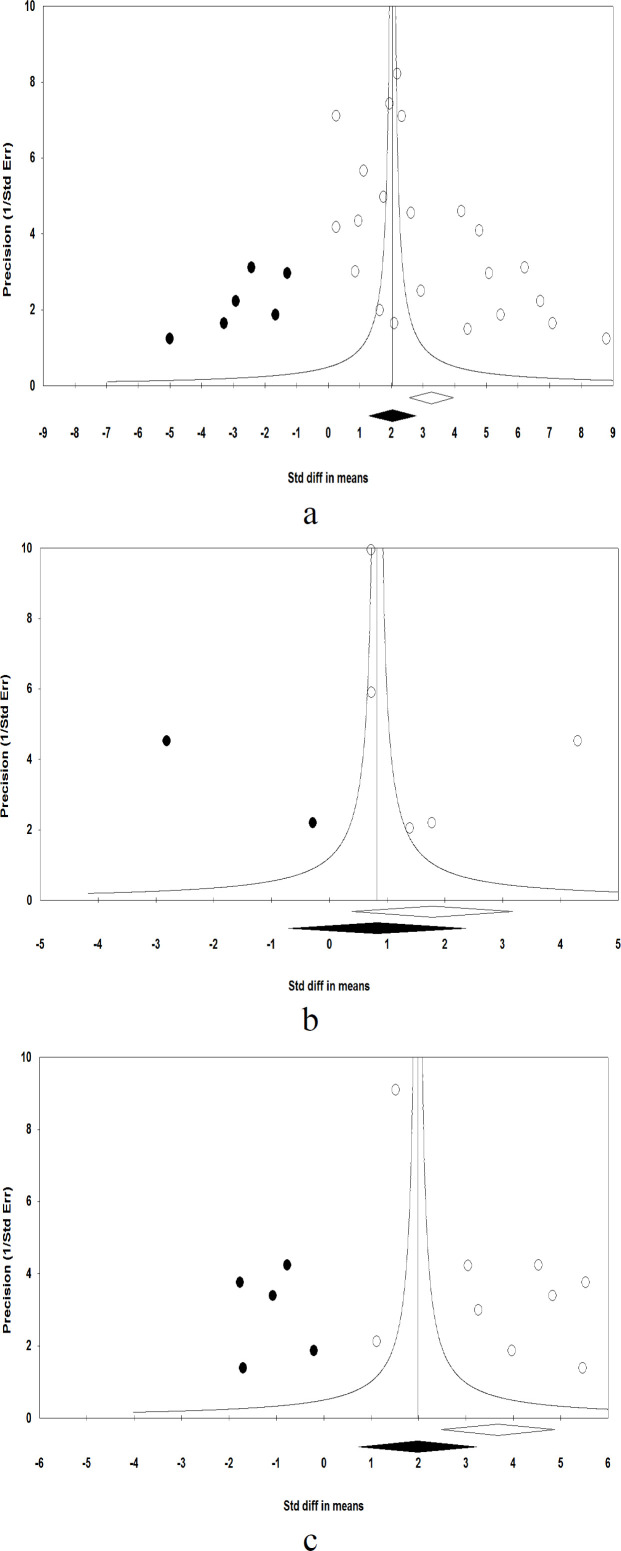
Random-effects Funnel plot detailing publication bias in the studies investigating the association between circulating levels of a) CRP, b) TNF-  and c) IL-6 with severity of n-COVID-19 in hospitalized patients after trimming and filling. Open circles represent observed published studies; closed circles represent imputed unpublished studies

Begg’s test for (CRP; *p*=0.07, TNF-; *p*=0.46 and IL6; *p*=0.91) and Egger’s test for (TNF-; *p*=0.45 and IL6; *p*=0.07) were not significant except for CRP with Egger’s test (*p*=0.01). Funnel plots for the outcomes of CRP, TNF-, and IL-6 were symmetrical, suggesting no potential publication bias. When the effect size of small-study bias was estimated using the trim-and-fill function, the addition of 6, 2, and 5 theoretically missing studies decreased the summary SMD to (2.03 mg/L; 95% CI 1.2, 2.7), (0.74 ng/mL; 95% CI 0.60, 0.88), and (1.9 ng/mL; 95% CI 0.73, 3.2) for CRP, TNF-, and IL-6, respectively, with the random-effects model (Fig. 5).

Sensitivity analyses indicated that the levels of CRP [SMD = 3.1 mg/L; (95% CI 2.4, 3.8); *p*<0.05], TNF- [SMD = 2.05 ng/mL; (95% CI -0.03, 4.1); *p* = 0.05], and IL-6 [SMD = 3.6 ng/mL; (95% CI 2.3, 4.9); *p*<0.05] were greater when studies with significant methodological limitations (NOS≤ 7) or studies that used large patient sample sizes were excluded. 

## Discussion

The results of previous studies have indicated that elevated levels of inflammatory cytokines in serum are related to liver and pulmonary damage in COVID-19 infection. The present study evaluated the monitoring value of the inflammatory marker levels for screening severe cases of COVID-19 in hospitalized patients. All observational studies were included in this meta-analysis. Here, increases in CRP, IL-6, and TNF-α levels in severe cases compared to mild cases of COVID-19 were revealed. This result suggests that elevated inflammatory cytokines are the most significant feature and predictive factor of severe COVID-19 that lead to liver damage in these patients. Based on previous studies, apart from the virus’s direct attack on liver tissue, systemic inflammation is the most important cause of liver damage in COVID-19 patients.

C-reactive protein (CRP) is found in blood plasma, and its circulating concentrations rise in response to inflammation. It is an acute-phase protein of hepatic origin that increases following interleukin-6 secretion by macrophages and T cells. It is synthesized by the liver in response to factors released by macrophages (36). The evidence suggests that C-reactive protein (CRP) levels are increased significantly by COVID-19 due to the inflammatory reaction. One hypothesis for this elevation is the overproduction of pro-inflammatory cytokines which attack the virus, but when this system becomes hyperactive, it can cause tissue damage, especially to the liver (37, 38). Furthermore, CRP is one of the key factors needed to diagnose and follow up the treatment of the novel coronavirus (Covid-19). Recent studies have shown that CRP levels can be used in the early diagnosis of pneumonia (39), and patients presenting with severe pneumonia had high CRP levels upon SARS-CoV-2 infection. Recently, Wang showed that CRP levels are positively correlated with the diameter of lung lesions and could reflect disease severity (40). Another study demonstrated that the mean level of CRP is higher in severe COVID-19 patients compared to mild cases. The results of the current meta-analysis indicated that the CRP level is positively correlated with the severity of COVID-19. 

Among various factors, TNF-α and IL-6 are two cytokines released during inflammation and are major mediators in several diseases. A recent study showed a systemic up-regulation of IL-6 during the acute phase of infection (41). This up-regulation suggested a potential association between IL-6 levels and virus virulence. Therefore, one of the main roles of IL-6 is to protect immunocompetence, defined as the ability of a host to respond to infections (42). While several investigations have reported the critical role of IL-6 in mounting a proper immune response during viral infections, other studies have linked this cytokine to increased disease severity. These findings reinforce the hypothesis that increased levels of IL-6 during viral infections possibly promote virus survival and exacerbation of the disease (43). IL-6 is a pleotropic cytokine produced in response to tissue damage in several disease and infections (44). Interleukin-producing cell types include fibroblasts, keratinocytes, mast cells, macrophages, and dendritic cells, and T and B cells are associated with the production of this cytokine (45). In addition to modulating the host immune response, IL-6 has been involved in the progression of various viral diseases. It is considered one of the most important cytokines during an infection, along with tumor necrosis factor alpha (TNF-α) (46). The evidence suggests that IL-6 plays an important role in the survival of the influenza virus in mice infected with this virus (47). Considering the evidence for the key role of inflammatory markers in exacerbating viral diseases and because COVID-19 is also a viral infectious disease, it can be concluded that the up-regulation of inflammatory cytokines (including CRP, IL-6, and TNF-α) can lead to the progression of the disease to the severe form and eventually cause liver damage in these patients.

Based on this analysis of the primary COVID-19 data, elevated levels of CRP, TNF-α, and IL-6 were found to be associated with increased odds of the severe form of COVID-19 that can result in liver damage in these cases. In this regard, it is hypothesized that pro-inflammatory marker levels may be related to the degree of liver damage with increased severity of COVID-19. 

Several limitations to this meta-analysis should be considered. First, meta-analyses are greatly influenced by the small sample size of studies included. Second, all the included articles summarized results from observational studies in China.

In conclusion, it is suggested that the incidence of increased systemic inflammation associated with liver damage is higher in patients with severe forms of COVID-19 infection. This finding may help clinicians in identifying the degree of liver damage in severe cases of COVID-19 with poor prognoses at an early stage.
